# A microfluidic transistor for automatic control of liquids

**DOI:** 10.1038/s41586-023-06517-3

**Published:** 2023-10-25

**Authors:** Kaustav A. Gopinathan, Avanish Mishra, Baris R. Mutlu, Jon F. Edd, Mehmet Toner

**Affiliations:** 1 BioMEMS Resource Center, Center for Engineering in Medicine and Surgical Services, Massachusetts General Hospital, Boston, MA USA; 2 Cancer Center, Massachusetts General Hospital, Boston, MA USA; 3 Department of Surgery, Massachusetts General Hospital, Boston, MA USA; 4 Shriners Children’s, Boston, MA USA

**Keywords:** Fluidics, Microfluidics, Lab-on-a-chip

## Abstract

Microfluidics have enabled notable advances in molecular biology^1,2^, synthetic chemistry^3,4^, diagnostics^5,6^ and tissue engineering^7^. However, there has long been a critical need in the field to manipulate fluids and suspended matter with the precision, modularity and scalability of electronic circuits^8–10^. Just as the electronic transistor enabled unprecedented advances in the automatic control of electricity on an electronic chip, a microfluidic analogue to the transistor could enable improvements in the automatic control of reagents, droplets and single cells on a microfluidic chip. Previous works on creating a microfluidic analogue to the electronic transistor^11–13^ did not replicate the transistor’s saturation behaviour, and could not achieve proportional amplification^14^, which is fundamental to modern circuit design^15^. Here we exploit the fluidic phenomenon of flow limitation^16^ to develop a microfluidic element capable of proportional amplification with flow–pressure characteristics completely analogous to the current–voltage characteristics of the electronic transistor. We then use this microfluidic transistor to directly translate fundamental electronic circuits into the fluidic domain, including the amplifier, regulator, level shifter, logic gate and latch. We also combine these building blocks to create more complex fluidic controllers, such as timers and clocks. Finally, we demonstrate a particle dispenser circuit that senses single suspended particles, performs signal processing and accordingly controls the movement of each particle in a deterministic fashion without electronics. By leveraging the vast repertoire of electronic circuit design, microfluidic-transistor-based circuits enable fluidic automatic controllers to manipulate liquids and single suspended particles for lab-on-a-chip platforms.

## Abstract (web summary)

Flow limitation is exploited to develop a microfluidic device exhibiting flow–pressure behaviour analogous to the current–voltage characteristics of an electronic transistor.

## Main

Given the great success of electrical processing systems, a long-standing goal in the field of microfluidics has been the creation of a fully integrated, automatic liquid processing system, sometimes termed a lab-on-a-chip^17,18^. Such a device should be capable of automatic control, which in microfluidics entails measuring, processing and generating controlled fluidic signals (pressure and flow state variables) to precisely manipulate microscopic samples.

Today, the most widespread technology to perform automatic control is the electronic circuit. With the invention of the vacuum tube and later, the transistor, electronic circuits were used to build stable automatic controllers with excellent precision and speed using negative feedback loops^15,19^. This feedback control was possible owing to the transistor’s defining ability to proportionally amplify signals (that is, to produce an output signal with the same shape as an arbitrary input signal, but at a higher amplitude)^14^. This capability led to an explosion of transistor-based analog and digital circuit designs, which culminated in the ultimate automatic control system: the electronic microprocessor. A microfluidic element with fluidic amplification capability analogous to that of the electronic transistor could likewise enhance the level of precision, speed and automatic control over fluidic signals and also enable the direct translation of the transistor-based electronic design repertoire towards the processing of biological and chemical samples.

The key advance of this study is to exploit the fluidic phenomenon of flow limitation to create a microfluidic element with the ability to perform proportional amplification of fluidic signals. As this element replicates all of the electronic transistor operating regimes (linear, cutoff and saturation) in the fluidic domain, we term it a microfluidic transistor. After characterizing this microfluidic transistor, we demonstrate that this single element enables one-to-one conversion of all three fundamental transistor circuit topologies and a wide range of classic electronic building blocks into the microfluidic domain including the amplifier, regulator, level shifter, NOT–AND (NAND) gate and set–reset (SR) latch. These circuit blocks enable processing of fluidic signals on-chip without external controllers.

We then cascade several of these building blocks together in more complex circuits such as automatic timers and fluidic clocks. Finally, we combine the signal-processing capabilities of the transistor-based circuits with the physical sample manipulation abilities of microfluidic traps to create an autonomous particle dispenser. This dispenser demonstrates, as a proof-of-concept, the basic aspects of a fully autonomous lab-on-a-chip system that uses fluidic controller circuitry to detect, manipulate and process individual physical samples. We configure this fluidic system to automatically perform deterministic single-particle ordering and concentration without any external optical or electronic components.

**Fig. 1:**
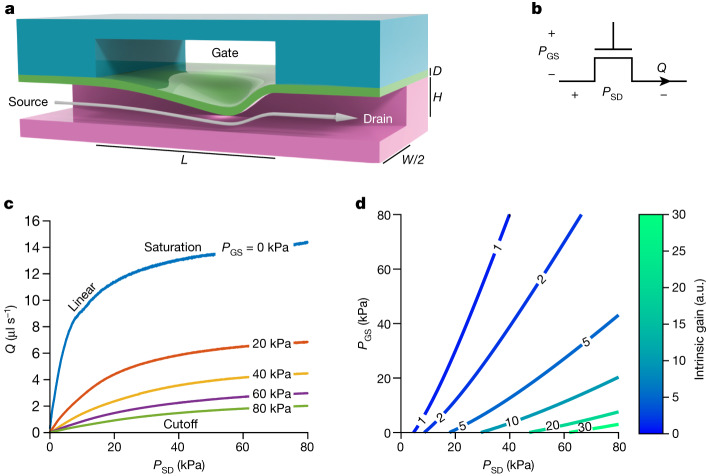
Analogous to an electronic transistor, elastomeric channels exhibit pressure-controlled flow limitation. **a**, Longitudinal section of the microfluidic transistor, fabricated from two layers of thick elastomer with channels (magenta, teal) and a thin elastomer membrane (green). Pressure applied between the gate and the source deflects the membrane, restricting flow (arrow) from the source to the drain in a nonlinear fashion known as flow limitation. **b**, Schematic symbol for the microfluidic transistor. The pressure difference between the gate and the source is *P*_GS_, and the pressure difference between the source and the drain is *P*_SD_. Volumetric flow through the drain is *Q*. **c**, Experimentally measured characteristic curves of the microfluidic transistor, demonstrating all three operation regimes seen in electronic transistors (linear, cutoff and saturation). **d**, Contour plot of the intrinsic gain of the microfluidic transistor as a function of *P*_GS_ and *P*_SD_, depicting a large region with intrinsic gain greater than one. a.u., arbitrary units.

The microfluidic transistor consists of two crossed channels of liquid separated by a deformable membrane (Fig. 1a) and is fabricated entirely from elastomer using standard soft-lithography techniques (Methods). It is represented schematically in Fig. 1b. When a pressure difference *P*_SD_ is applied between the source and drain terminals, the membrane between the crossed channels deforms. With carefully chosen geometry, this self-deformation limits volumetric flow *Q* passing through the drain in a particular nonlinear manner known as flow limitation^16^, which is key to the transistor’s amplification capability. The extent of this flow limitation effect can be modulated by applying a pressure *P*_GS_ between the gate and source terminals.

**Extended Data Fig. 1:**
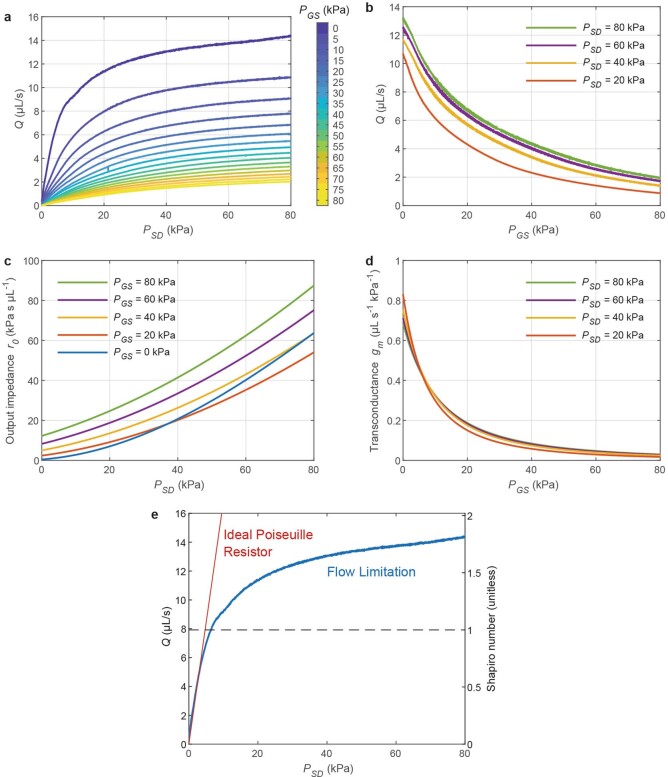
Additional characterization of a microfluidic transistor. **a**, Experimentally measured characteristic output curves for a single transistor. At large values of *P*_*SD*_, the device exhibits a saturation-like phenomenon due to flow limitation. The flow rate can be modulated by applying a pressure *P*_*GS*_. **b**, Transfer characteristics for a microfluidic transistor. The high slopes of the curves at low *P*_*GS*_ indicate that the device has a high transconductance suitable for analog amplification. **c**, Output impedance for a microfluidic transistor. The output impedance is large at high values of *P*_*SD*_ due to the phenomenon of flow limitation taking place. **d**, Transconductance for a microfluidic transistor. The strong overlap of the curves demonstrate good output impedance, and the high transconductance values at low *P*_*GS*_ lead to high intrinsic gain. **e**, Microfluidic transistors exhibit flow limitation as the Shapiro number exceeds one. In the regime where the Shapiro number is less than one, the flow-pressure characteristics of the microfluidic transistor (blue) follow the linear relationship predicted by the Poiseuille equation (red). As the Shapiro number exceeds one (dashed line), the flow-pressure characteristics deviate, and the system exhibits flow limitation.

The microfluidic transistor is characterized analogously to the electronic p-channel junction field-effect transistor. Figure 1c provides the characteristic curves for a microfluidic transistor with dimensions provided in Extended Data Table 1. Volumetric flow *Q* is recorded while *P*_SD_ is swept across a range of pressures and *P*_GS_ is held at fixed values, resulting in the fluidic version of the classic transistor characteristic curves. The flow limitation effect causes the characteristic curves to level off at high *P*_SD_, akin to the saturation behaviour of the electronic transistor. The complete set of characteristic curves for additional values of *P*_GS_ is provided in Extended Data Fig. 1a. The transfer characteristics, output impedance and transconductance plots for the microfluidic transistor are provided in Extended Data Fig. 1b–d.

The function-defining characteristic of any transistor is its ability to proportionally amplify signals^20^. This is quantified by its intrinsic gain *A*_0_, a dimensionless measure of the maximum proportional amplification achievable for a given set of potentials applied across the source, gate and drain^14^ (derivation in Methods). Crucially, for a microfluidic element to amplify like a transistor, there must be a practically achievable range of values for *P*_SD_ and *P*_GS_ for which the intrinsic gain is greater than one. Figure 1d shows a contour plot of the intrinsic gain as a function of applied *P*_GS_ and *P*_SD_, computed using the characterization data of Extended Data Fig. 1a. The contour plot reveals a large operating region where the intrinsic gain is much greater than one, indicating that this microfluidic element is capable of proportionally amplifying signals and thus functions like a transistor.

These high intrinsic gains were achieved by exploiting the phenomenon of flow limitation. This phenomenon is observed in certain confined flows through tubes with deformable boundaries (including the human vena cava), for which increasing the pressure drop across the tube beyond a threshold does not substantially increase the flow rate through the tube^16,21^. Flow limitation occurs in systems for which the dimensionless Shapiro number *S* is greater than one (that is, when the characteristic velocity of the fluid exceeds a characteristic speed of a pressure wave travelling through the system)^22^. For the microfluidic channels considered here, the Shapiro number is given by (derivation in Methods):

$$S=Q{\left(\frac{{A}^{3}}{\rho }\frac{{{\rm{\pi }}}^{4}E{D}^{3}}{6{W}^{5}\left(1-{\nu }^{2}\right)}\right)}^{-\frac{1}{2}}$$

in which *Q* is flow rate, *ρ* is fluid density, *A* is channel cross-sectional area, *W* is channel width, *D* is membrane thickness, *E* is membrane Young’s modulus, and *ν* is membrane Poisson ratio. Using the above equation (valid when *P*_GS_ *=* 0) and the measurements from Fig. 1c, we verify that when the Shapiro number exceeds one, the flow–pressure characteristic of the transistor diverges from the typical linear Poiseuille behaviour and enters flow limitation (Extended Data Fig. 1e). This may also be observed in the saturation region of Fig. 1c where the flow–pressure curves become nearly flat. Although the curves show a slight upwards tilt in the saturation region due to the leakage of fluid through the corners of the channel, this finite output impedance (Extended Data Fig. 1c) still produces an intrinsic gain greater than 20 and therefore does not substantially affect transistor function. The flow limitation effect observed in the microfluidic transistor is strikingly similar to the saturation behaviour of the field-effect transistor, and these effects are fundamental to how each device achieves a high intrinsic gain and performs amplification.

**Fig. 2:**
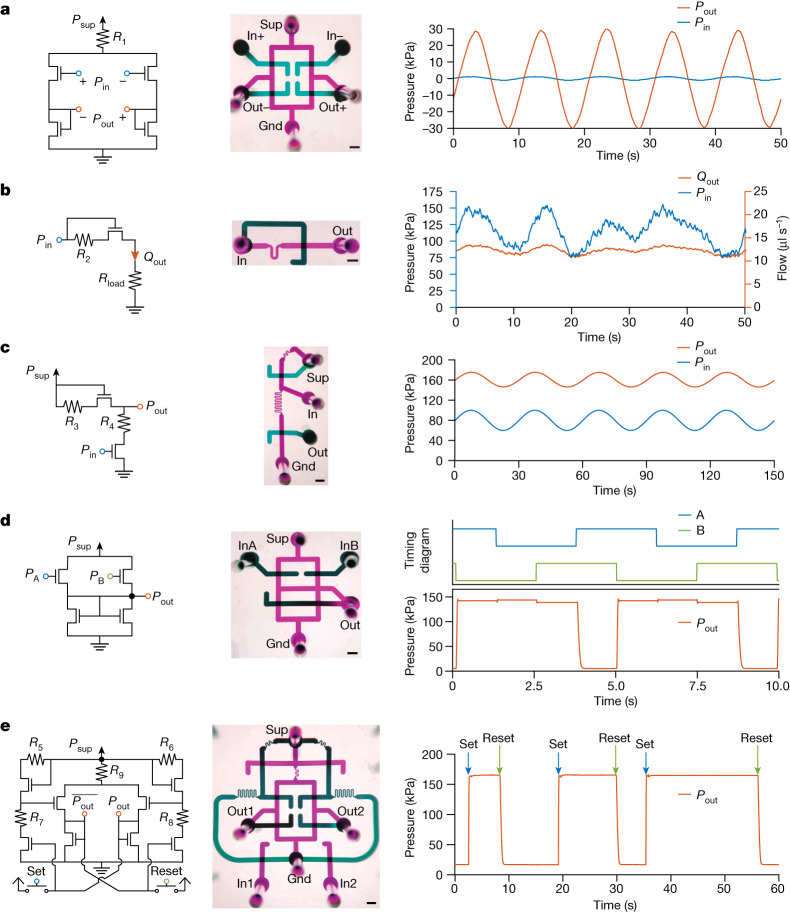
Microfluidic-transistor-based circuits replicate the behaviour of key electronic circuits. For each circuit, the schematic diagram (left), a photo of the microfluidic implementation with ports labelled (middle; false colour; scale bars, 1 mm) and a representative demonstration of circuit function (right) are provided. Microfluidic port labels are defined and detailed in the Methods and Extended Data Figs. 7 and 8. Power supply and ground ports are labelled ‘Sup’ and ‘Gnd’, respectively. **a**, A fluidic differential amplifier. The input differential pressure signal (blue, applied at ‘In+’ and ‘In–’) is amplified with a gain of 22 to generate the output differential signal (orange, measured at ‘Out+’ and ‘Out–’). **b**, A flow regulator. The input pressure (blue, applied at ‘In’) varying from 75 to 150 kPa is regulated to supply a target flow (orange, measured at ‘Out’) of 12 ± 1.5 μl s^−1^ to a load. **c**, A level shifter. The baseline of a varying input pressure signal (blue, applied at ‘In’) is shifted up by 80 kPa to produce an output pressure signal with the same morphology (orange, measured at ‘Out’). **d**, A NAND gate. The output signal (orange, measured at ‘Out’) is low only if both input signals (blue and green, applied at ‘InA’ and ‘InB’) are high. **e**, An SR latch. The persistent state of the latch (orange, measured at ‘Out2’, complement state measured at ‘Out1’) can be set to high or low pressure based on transient pulses applied to ‘set’ (blue) or ‘reset’ (green) the input ports ‘In1’ or ‘In2’ high or low.

**Extended Data Fig. 2:**
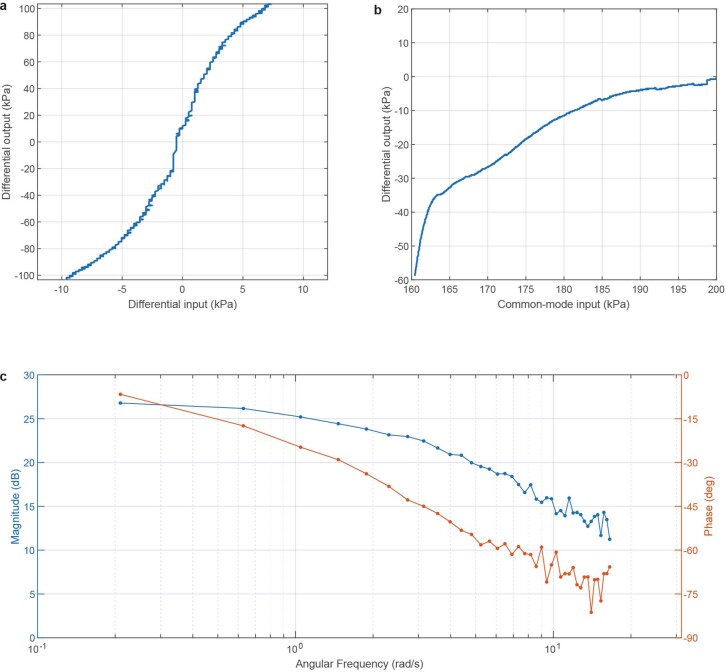
Additional characterization of the differential amplifier. **a**, Distortion (transfer) characteristics of the differential amplifier. The differential amplifier provides relatively linear amplification for amplitudes below 5 kPa. As in electronics, application of negative feedback can further linearize this output^15^. **b**, Common-mode rejection of the differential amplifier. The differential output is less sensitive to common-mode changes when biased around 190 kPa. **c**, Frequency response of the differential amplifier. Fourier analysis of the first 40 odd-harmonics of a square wave input show a low-frequency gain of approximately 27 dB.

**Extended Data Fig. 3:**
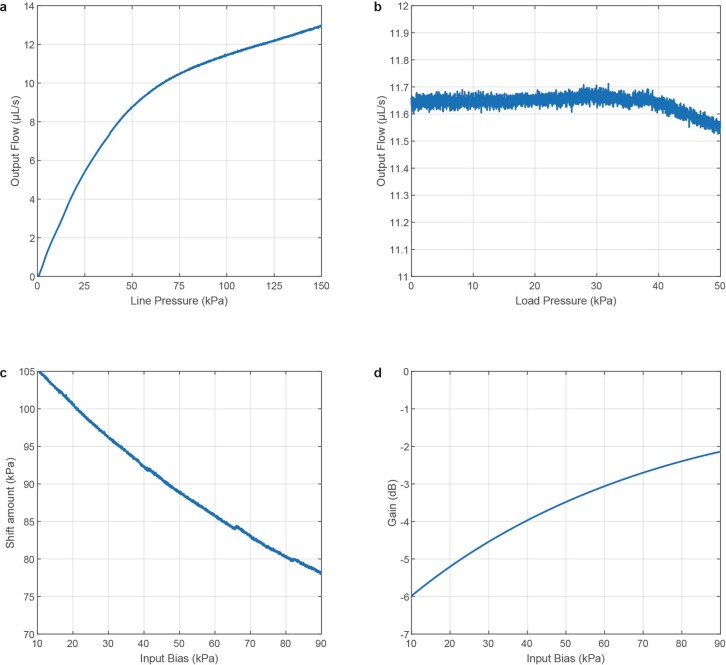
Additional characterization of the flow regulator and the level shifter. **a**, Line regulation of the flow regulator through a 2 kPa s μl^−1^ load. The output flow is less sensitive to changes to the line pressure above 75 kPa. **b**, Load regulation of the flow regulator. With a line pressure of 100 kPa, the output flow is insensitive to changes in the load up to 50 kPa. **c**, Pressure shift of the level shifter. The level shifter is capable of shifting signals by over 80 kPa. **d**, Gain of the level shifter. Depending on the bias, there is a small drop in the amplitude of output signal. Note that since this circuit operates in a common-drain configuration, the gain in decibels is expected to be negative.

**Extended Data Fig. 4:**
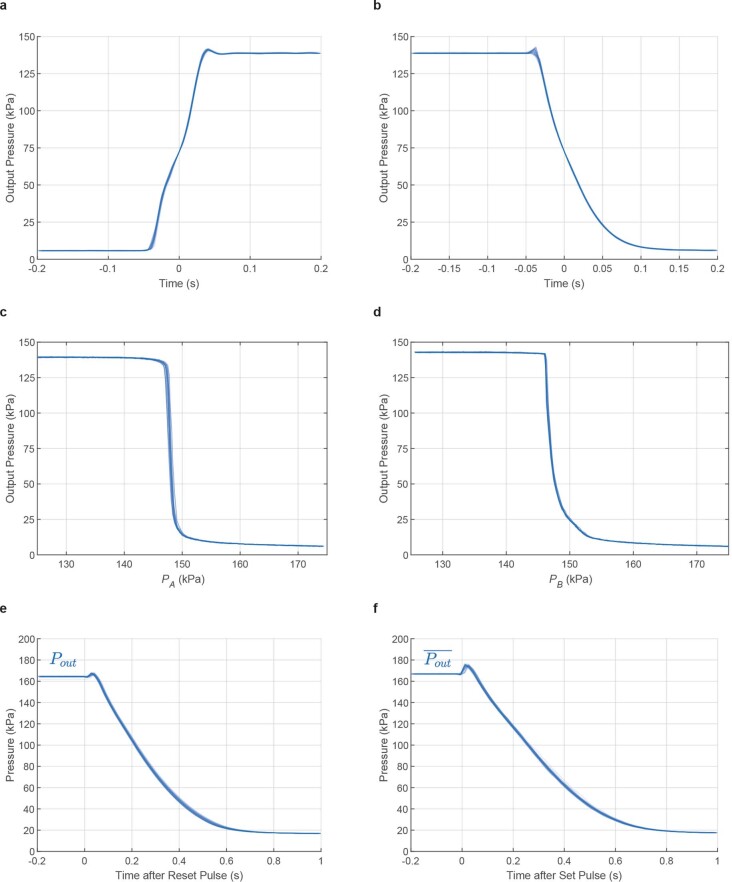
Additional characterization of the NAND gate and the SR Latch. **a**,**b**, Output transition and dynamics of the NAND gate. Input signal B was set to high while input signal A was toggled between high and low, causing the output to toggle between low and high. The transitions for n = 55 individual rising (**a**) and falling (**b**) output edges are overlaid, showing rise and fall times of less than 100 ms with good repeatability. **c**,**d**, Transfer characteristics of the NAND gate. **c**, Input A was swept across a range of pressures while input B was held high, and the output signals for n = 10 sweeps were overlaid. **d**, Input B was then swept while input A was held high, and the output signals for n = 10 sweeps were overlaid. The sharp transitions between logic levels indicate a large noise margin for digital signals. **e**, Reset response dynamics of the SR-latch. After initializing the latch to a high output state (*P*_*out*_ high and ${\bar{P}}_{{out}}$ low), a reset pulse flipped the output *P*_*out*_ low within 800 ms. The overlaid output for n = 60 such events demonstrates repeatability. **f**, Set response dynamics of the SR-latch. After initializing the latch to a low output state (*P*_*out*_ low and ${\bar{P}}_{{out}}$ high), a set pulse flipped the complementary output ${\bar{P}}_{{out}}$ low within 800 ms. The overlaid output for n = 60 such events demonstrates repeatability.

To illustrate the flexibility of the microfluidic transistor, we demonstrate microfluidic analogues to key electronic circuit blocks (Fig. 2). These five circuit blocks were specifically chosen to be fundamental circuits commonly used across analog and digital electronics. The three building blocks chosen from analog circuit design—the amplifier, regulator and level shifter—exemplify all three fundamental topologies of the field-effect transistor: common source, common gate and common drain, respectively^14^. The two building blocks chosen from digital circuit design—the NAND gate and latch—demonstrate digital logic and memory, respectively^20^. For each circuit, we provide characterization studies to evaluate performance, similar to the studies typically found in electronic data sheets (Extended Data Figs. 2–4). The specific circuit component values are provided in Extended Data Table 1. Owing to the relatively simple crossing-channel design of the microfluidic transistor (once the specific flow limitation geometry is determined from the Shapiro equation), all data shown in Fig. 2 were from the first or second chip fabricated. Pinout diagrams and setup for each circuit are provided in Extended Data Figs. 7 and 8. Although it is possible to use other microfluidic techniques to individually accomplish the same functions as the regulator, NAND gate and latch circuits shown here (Extended Data Table 2), other approaches were unable to demonstrate all three transistor topologies with a single platform. By demonstrating these topologies in Fig. 2, our technology in principle enables microfluidic signal processing operations beyond digital logic and opens up the extensive analog and digital design repertoire of transistor-based electronics for microfluidic replication.

As amplification is the defining characteristic of a transistor^20^, we first demonstrate microfluidic transistors in a differential amplifier exemplifying the common-source topology (Fig. 2a). This analog circuit amplifies an input differential pressure signal by a gain of over 20. Advanced characterization studies for this circuit, including the frequency response, common-mode rejection and distortion, are provided in Extended Data Fig. 2. Amplifiers are the fundamental building blocks of analog circuits, used ubiquitously in signal processing and feedback control^14,15^.

A flow regulator is demonstrated in Fig. 2b exemplifying the common-gate topology. This analog circuit supplies a constant output flow to a downstream load regardless of the input pressure level. Advanced characterization studies for this circuit, including the load and line regulation, are provided in Extended Data Fig. 3a,b. Regulators may be used to run microfluidic devices using unregulated pressure sources in resource-limited settings.

A level shifter is demonstrated in Fig. 2c exemplifying the common-drain topology. This analog circuit translates the baseline pressure of the input signal to a higher output baseline pressure without affecting the signal morphology. Advanced characterization studies for this circuit, including the shift amount and gain, are provided in Extended Data Fig. 3c,d. Level shifters allow multiple circuit blocks to be cascaded sequentially, even if they require different biasing pressures, enabling design modularity.

A NAND gate is demonstrated in Fig. 2d. This digital logic gate produces a low output pressure only if both inputs are at a high pressure. NAND gates are universal logic gates, so can be combined to implement all other Boolean logic operations for general digital signal processing. Advanced characterization studies for this circuit, including the output dynamics and transfer characteristics, are provided in Extended Data Fig. 4a–d. Logic gates may be used to synchronize fluidic events or compute binary arithmetic.

An SR latch (bistable multivibrator) is demonstrated in Fig. 2e. This digital circuit has two stable output states that can be set high or low persistently after receiving a transient ‘set’ or ‘reset’ pressure pulse, and therefore hold memory. Advanced characterization studies for this circuit, including the response dynamics, are provided in Extended Data Fig. 4e,f. Cascaded latches act as fluidic memory and can store binary numbers. Therefore, they may be used to count fluidic events or perform sequential combinatorial operations that require memory of the circuit’s previous state.

**Fig. 3:**
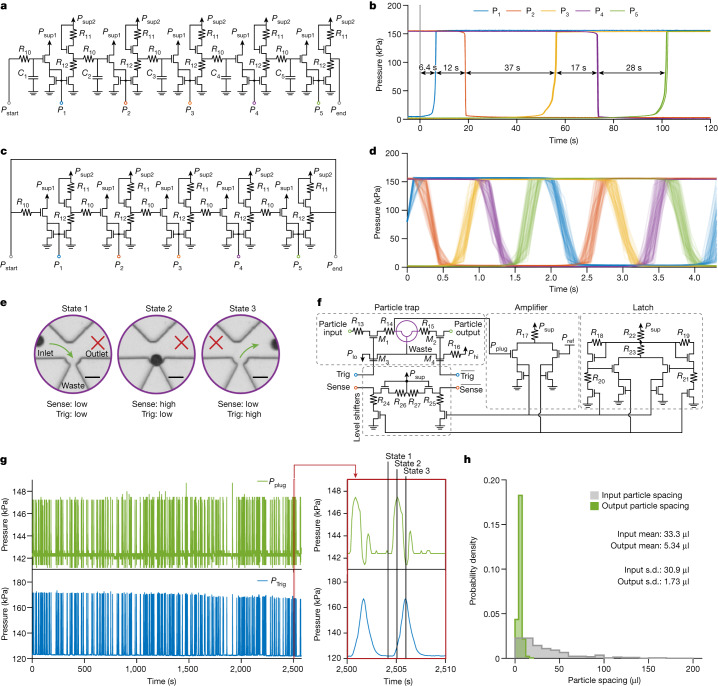
Microfluidic transistors enable timing operations and smart dispenser circuits for autonomous single-particle manipulation. **a**, Circuit schematic for a sequential delay timer, comprising many cascaded low-pass filters, amplifiers and level shifters. **b**, A step signal applied to *P*_start_ at time *t* = 0 propagates through the circuit blocks, generating controllable time intervals to trigger sequential fluidic events. Capacitance values were selected to time out five fluidic events sequentially with differing intervals. Triggered pressure signals for three trials of this circuit are overlaid and plotted with mean interval duration shown. **c**, Circuit schematic for a ring oscillator comprising five amplifiers and five level shifters. **d**, The ring oscillator spontaneously generates square waves at the output of each inverter, separated in phase by a fifth of a period. The oscillator signals were measured for several minutes, and 63 unit intervals of each signal are overlaid and plotted in the eye diagram. **e**, Overview of the smart dispenser operation, depicting the core microfluidic trap in different states as it senses and dispenses a single particle (scale bars, 50 μm). **f**, Circuit schematic of the smart dispenser comprising several circuit blocks and the microfluidic trap (purple). **g**, Deterministic single-particle ordering and concentration using the smart dispenser. This dispenser configuration has the Trig and Sense lines directly connected, so that individual particles are sensed and immediately dispensed into the output channel. Pressure signals from the trap itself (*P*_plug_) and the trigger (*P*_Trig_) for a run of *n* = 230 particles are shown, along with a representative dispense event to observe the individual dynamics (red inset). **h**, Histograms of input and output particle spacing when using the smart dispenser in this configuration, showing a 6-fold drop in the spacing mean (indicating particle concentration) and a 17-fold drop in the spacing standard deviation (indicating particle ordering).

Next we demonstrate how the building blocks of Fig. 2 may be cascaded together to form more complex circuits. Figure 3a,b depicts a sequential delay timer, which can be used to time out sequential fluidic events. It is constructed by cascading a series of inverters separated by low-pass filters (Fig. 3a). Each inverter consists of single-input amplifier and level shifter blocks. A step signal (Start) is delayed for a fixed time period by the first low-pass filter before activating the subsequent inverter. The signal then moves to the next low-pass filter, which again produces a fixed delay, and the signal gradually propagates through as many steps as required by the application. The time intervals between each step can be adjusted by altering the resistance or capacitance of the filter before the inverter. Figure 3b demonstrates the fluidic timer timing out five events sequentially with a variety of timing intervals. The data shown are three trials of this circuit superimposed, showing good repeatability for the timing intervals across several trials.

**Extended Data Fig. 5:**
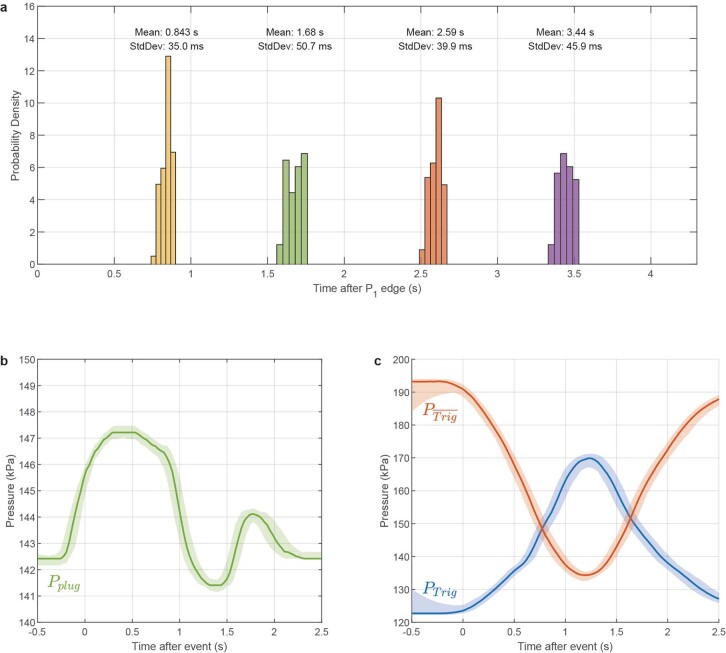
Additional characterization of complex circuits combining several building blocks. **a**, Jitter plot of the ring oscillator circuit. The time intervals between the rising edges of the *P*_1_ clock signal and the subsequent clock signals are plotted in a histogram to measure the clock jitter over 63 periods .The mean intervals are equally spaced by one-fifth of the clock period. **b**, Collated plug signals for individual events of the smart particle dispenser. After a particle is trapped, the pressure upstream of the trap (*P*_*plug*_) rises in a repeatable fashion. The *P*_*plug*_ pressure signal for n = 230 trapping events were aggregated, and a pointwise median signal with 90% and 10% quantile bands were plotted. **c**, Collated trigger signals for individual events of the smart particle dispenser. The amplifier and the level shifters process the *P*_*plug*_ signal to produce the trigger signals *P*_*Trig*_ and ${P}_{\overline{{Trig}}}$. These signals for n = 230 trapping events were aggregated, and a pointwise median signal with 90% and 10% quantile bands are plotted.

We then removed the capacitances and fed back the output of the last inverter to the input of the first inverter to create a ring oscillator (Fig. 3c). To evaluate signal quality, we provide an eye diagram of the inverter outputs with 63 overlaid periods clocked by the first inverter (Fig. 3d). Further analysis and quantification of the clock jitter is provided in Extended Data Fig. 5a. The oscillator and the timer circuits (five amplifiers, five level shifters and five low-pass filters) also demonstrate how multiple building blocks from Fig. 2 may be combined in a straightforward fashion for more complex operations. These circuits may be applied to synchronize and time out sequential fluidic events, such as for executing multi-step chemical protocols.

Although the circuits of Fig. 2 demonstrate how the microfluidic transistor can be used to replicate the main building blocks of electronics, we also sought to demonstrate a proof-of-concept application for the microfluidic transistor that cannot be performed by an electronic transistor: directly detecting and processing physical objects suspended in liquid. Figure 3e–h demonstrates a ‘smart’ particle dispenser capable of detecting and programmatically dispensing individual suspended particles. At the core of the dispenser is a microfluidic particle trap with an inlet, outlet and waste channel (Fig. 3e). Normally, with no particle in the trap, fluid flows directly from the inlet to the waste channels (state 1). When a particle becomes trapped, the dispenser detects its presence by amplifying the slight rise in upstream pressure *P*_plug_ and produces a high Sense pressure signal, indicating that it is holding a trapped particle and is awaiting the trigger signal to dispense it (state 2). If the dispenser then receives a high ‘Trig’ pressure signal, the flow through the trap is reversed and the particle is ejected into the outlet channel (state 3). The dispenser then returns to its initial state to process a new particle. To perform this complex sequence of dispensing operations, several signal processing circuit blocks from Fig. 2 including the amplifier, level shifter and latch, are utilized in the dispenser’s control circuitry (Fig. 3f). Component values and circuit details are provided in Extended Data Table 1.

Different circuit configurations of this autonomous dispenser block offer utility for counting, ordering, encapsulating and distributing individual particles or potentially biological cells. Here we demonstrate a simple configuration of the dispenser by connecting the ‘Sense’ and ‘Trig’ lines in a feedback loop. This configuration results in deterministic particle ordering and concentration in the output channel, as demonstrated in Fig. 3g using 40-μm polystyrene beads. Although particles enter the dispenser spaced randomly longitudinally (as a Poisson process)^23^, the particles exit following a tight distribution of equal spacing along the output stream (Fig. 3h and Extended Data Fig. 5b,c). The 6-fold drop in spacing mean and 17-fold drop in spacing standard deviation indicate that this configuration of the dispenser circuit block successfully concentrated and ordered the particles.

It is important to note that all of the signal processing, particle manipulation and automatic control demonstrated here were performed entirely in the microfluidic domain through the use of amplifiers and other microfluidic-transistor-based circuit blocks, requiring only constant-pressure sources to supply power. Although there are other microfluidic techniques to order particles in channels, typically using inertial^24,25^ or viscoelastic^26^ phenomena, we have provided this circuit as a proof-of-concept to demonstrate microfluidic automatic control of physical samples using feedback. The dispenser circuit block may be readily configured with additional signal-processing circuitry on Sense and Trig to perform more complex particle dispensing tasks, and future improvements in transistor speed and performance will correspondingly increase the throughput of the particle manipulations performed by this circuit.

The transistor presented in this paper builds on previous work carried out to develop microfluidic valve systems for automatic control (Extended Data Table 2). One strategy used by some microfluidic systems to achieve this is to outsource the automatic control to external electronic systems, and interface this electronic controller with the microfluidic chip via pneumatically controlled microvalves^9^. This approach, driven by the development of low-dead-volume, pneumatically driven valves, has found broad utility in genomic platforms, and allows for programming via the electronic controller^27,28^. However, the separation of the electronic controller from the fluids and the additional communication interface between the two signal domains limits scalability and increases feedback delay^8,9,29^.

These issues were subsequently addressed by building a digital pneumatic controller integrated into the microfluidic chip itself^13,30^. At the heart of this controller is a valve where an input pneumatic digital signal can switch on or off a larger output pneumatic digital signal, analogous to the behaviour of the electronic relay. This switching capability is sufficient to build impressive pneumatic digital circuits, including binary logic^29–32^, latches^13,32^ and an 8-bit adder^30^. Further work^8,11,12,33^ has extended pneumatic valve systems to function with liquids instead of gases, resulting in the creation of liquid logic gates, oscillators and latches. Innovative chemofluidic transistors have also been developed that switch on the basis of chemical signals, using hydrogels that can be chemically stimulated to swell and block microfluidic channels, thereby switching flows on or off^34,35^. This technology has been used to create chemofluidic oscillators^36–38^, latches^37,39^ and digital logic circuits^37,38^ that function with chemical signals.

However, it is important to note that none of these existing valve systems for liquid control has demonstrated proportional amplification, in which an arbitrary input signal is converted to an output signal with the same shape at a higher amplitude^14^ (Extended Data Table 2). In electronics, this fundamental capability is what functionally separates the transistor from the electronic relay. Although it is possible to build digital logic, oscillators and latches without proportional amplification using only relays, these constitute only a subset of signal processing operations used in electronics. The amplification capability of the transistor was crucial for the analog half of circuit design, and digital circuits without proportional amplification suffer from an inability to apply negative feedback control with an error much smaller than the difference between the digital logic levels used^40^. This limitation is especially relevant for automatic control systems in microfluidics, for which the samples involved necessitate small signals and precise negative feedback control. For example, without proportional amplification, the tiny pressure signals generated by microscopic samples (for example, cells) are not strong enough on their own to be sensed and controlled via negative feedback by digital valve-based systems that typically switch with much higher logic levels (often tens of kilopascals)^12,13,30^. Additionally, digital logic systems typically require far more components and interconnects for practical operations than analog circuits utilizing proportional amplification^41,42^. This problem, colloquially known in electronics as the tyranny of numbers^43^, is particularly pertinent given that today’s microfluidic circuit elements are much larger than their electronic counterparts.

The microfluidic transistor described here is capable of proportional amplification with a large region of high intrinsic gain. It replicates all three transistor topologies from circuit theory (common-source, common-gate and common-drain) and is therefore suitable for implementing fluidic circuits from the vast repertoire of transistor-based analog and digital circuit designs of electronics. Microfluidic transistor-based circuits function without any external control pneumatics, electronics or optics. With the ability to both process fluidic signals and automatically control single particles on the basis of those signals, we predict that microfluidictransistor-based circuitry will unlock the breadth and depth of electronic circuit design to address the problem of automatic control for microfluidic lab-on-a-chip technologies.

## Methods

### Calculation of intrinsic gain

Although intrinsic gain was originally defined in the context of electronic transistors in terms of voltage and current^14^, we may follow an analogous derivation to define the intrinsic gain for a microfluidic transistor in terms of pressure and flow. For a microfluidic transistor for which the flow *Q* is a function of the pressures *P*_SD_ and *P*_GS_ applied across its terminals, the transconductance *g*_m_ is given by:

1 $${g}_{{\rm{m}}}=\frac{\partial Q}{\partial {P}_{{\rm{GS}}}}$$

and the output impedance *r*_o_ is given by:

2 $${r}_{{\rm{o}}}={\left(\frac{{\rm{\partial }}Q}{{\rm{\partial }}{P}_{{\rm{S}}{\rm{D}}}}\right)}^{-1}$$

Then the dimensionless intrinsic gain *A*_0_ is given by:

3 $${A}_{0}=|{g}_{{\rm{m}}}{r}_{{\rm{o}}}|=|\frac{{\rm{\partial }}Q}{{\rm{\partial }}{P}_{{\rm{G}}{\rm{S}}}}{\left(\frac{{\rm{\partial }}Q}{{\rm{\partial }}{P}_{{\rm{S}}{\rm{D}}}}\right)}^{-1}|$$

Note that this is analogous to the formula used in electronics for field-effect transistors, substituting pressure and flow for voltage and current^14^.

### Shapiro number in rectangular channels

In his seminal work describing flow limitation, Ascher Shapiro mathematically modelled the flow of an internal incompressible Newtonian fluid through a thin-walled deformable tube^16^. For this system, Shapiro defined a “characteristic wave propagation speed” *c* by the following:

4 $${c}^{2}=\frac{A}{\rho }\frac{{\rm{d}}{p}_{{\rm{t}}}}{{\rm{d}}A}$$

in which *A* is a characteristic cross-sectional area of the tube, and *ρ* is the fluid density. The term $\frac{{\rm{d}}{p}_{{\rm{t}}}}{{\rm{d}}A}$ couples structural deformation of the tube to the fluid flow. In previous studies, this term has been deduced on the basis of the ‘tube law’ for the system, which is the relationship between the cross-sectional area of the tube and the transmural pressure *p*_t_ across its walls. Typically, if the internal pressure of the tube is held constant, increasing the external pressure will cause the tube to deform and cause its cross-sectional area to drop.

Although the empirically derived tube law relationship was originally used to describe the deformation of thin-walled cylindrical tubes, here we consider the deformation of a square piece of thin membrane over a channel with a rectangular cross-section (Fig. 1a). The reciprocal hydraulic compliance of this membrane–channel fluidic system can be derived by plate theory as^11^:

5 $$\frac{{\rm{d}}{p}_{{\rm{t}}}}{{\rm{d}}V}=\frac{{{\rm{\pi }}}^{4}E{D}^{3}}{6{W}^{6}\left(1-{\nu }^{2}\right)}$$

in which *V* is the volume of fluid in the channel under the membrane, *W* is the characteristic length scale of the square membrane, *D* is the membrane thickness, *E* is the Young’s modulus of the membrane material, and *ν* is the Poisson ratio of the membrane material. Dividing both sides by the length of the square membrane, we obtain the following characteristic ‘tube law’ for a channel with a deformable square membrane:

6 $$\frac{{\rm{d}}{p}_{{\rm{t}}}}{{\rm{d}}A}=\frac{{{\rm{\pi }}}^{4}E{D}^{3}}{6{W}^{5}\left(1-{\nu }^{2}\right)}$$

Substituting this into equation (4), we obtain the following expression for the characteristic wave speed *c*:

7 $${c}^{2}=\,\frac{A}{\rho }\frac{{{\rm{\pi }}}^{4}E{D}^{3}}{6{W}^{5}\left(1-{\nu }^{2}\right)}$$

The Shapiro number *S* for this system is then simply the ratio of the characteristic fluid velocity to the characteristic wave speed of the channel. In terms of the flow rate *Q*, this is given by:

8 $$S=\frac{Q}{{Ac}}=Q{\left(\frac{{A}^{3}}{\rho }\frac{{\pi }^{4}E{D}^{3}}{6{W}^{5}\left(1-{\nu }^{2}\right)}\right)}^{-\frac{1}{2}}$$

For the microfluidic transistor characterized in Fig. 1c, the channel width *W* is 500 μm, the characteristic cross-sectional area *A* is 0.0275 mm^2^, the membrane thickness *D* is 20 μm, the membrane Poisson’s ratio *ν* is 0.5, the Young’s modulus *E* is 550 kPa, and the fluid density *ρ* is 1.01 g ml^−1^ (refs. ^44,45^). We may then use the characteristic curve measurements to compute the Shapiro number directly from the measured flow rate (Extended Data Fig. 1e). Note that in this analysis we consider only the curve for which *P*_GS_ *=* 0, which is the case analysed by Shapiro.

The Shapiro number delineates a critical transition in the behaviour of the membrane–channel system (Extended Data Fig. 1e). When the Shapiro number is much less than one, the deformation of the membrane does not substantially restrict flow, and the channel exhibits flow–pressure relationships as predicted by the Poiseuille equation. When the Shapiro number is greater than one, the deformation of the membrane substantially restricts flow, and the phenomenon of flow limitation takes place^22^.

As this analysis indicates a dependence of the Shapiro number on the channel height and membrane thickness, we tightly controlled the channel height using spin-coating of SU-8 and used pre-formed silicone membranes (Elastosil Film 2030 250/20, Wacker Chemie) when fabricating our chips using soft lithography.

### Microfluidic device fabrication

The photolithography masks for all devices presented in this work may be found in Supplementary Data 1. All devices used in this work were fabricated from two layers of polydimethylsiloxane (PDMS) and a thin silicone membrane (Fig. 1a). Standard soft-lithography techniques were used to fabricate each layer. In brief, SU-8 50 negative photoresist (Kayaku Advanced Materials) was spin-coated onto a silicon wafer at 2,450 r.p.m. for 30 s. The channels were patterned onto the SU-8 by exposing the wafer with 365 nm ultraviolet radiation through a photomask. The wafer was subsequently developed using Baker BTS-220 SU-8 developer to create the mould for the PDMS. For each device, two such moulds were made for the upper and lower PDMS layers. PDMS (Dow Sylgard 184 Kit, Ellsworth Adhesives) was prepared in a 6:1 ratio of base to crosslinker and poured into each mould to create a 4-mm-thick layer. The high ratio of crosslinker to base was used to minimize the deformation of the PDMS resistor channels as the channels were pressurized. The PDMS layers were cured in a convection oven for 20 h at 70 °C, and then cut and peeled from the mould.

**Extended Data Fig. 6:**
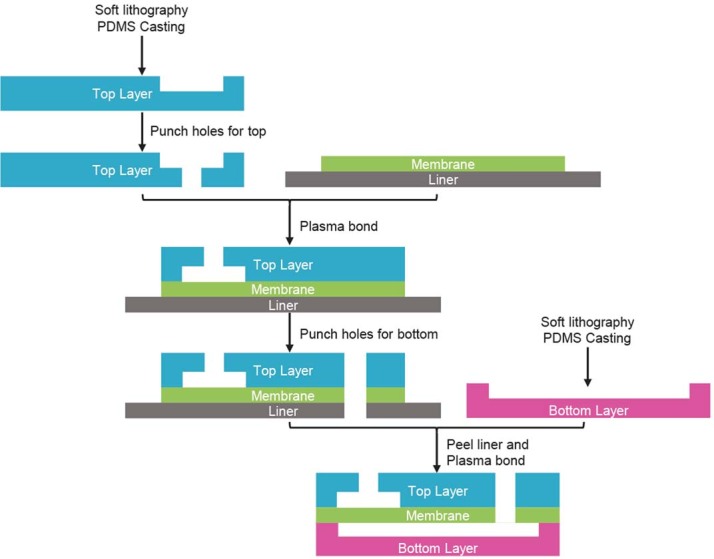
Assembly of multilayer microfluidic transistor-based circuits. Soft-lithography techniques are used to fabricate the top and bottom PDMS layers. Top layer ports are punched into the top PDMS layer. Then, the layer is bonded with a thin silicone membrane under oxygen plasma. Ports for the bottom layer are then punched into the top layer-membrane assembly. The assembly is aligned by hand and finally bonded with the bottom layer under oxygen plasma.

After casting the upper and lower layers of the device from PDMS, they were assembled to make the final microfluidic chips (Extended Data Fig. 6). A 1.2-mm biopsy punch was used to punch out appropriate ports in the upper PDMS layer. The PDMS layer was then bonded to a 20-μm-thick silicone membrane (Elastosil Film 2030 250/20, Wacker Chemie) by means of oxygen plasma treatment and baked at 80 °C for 15 min on a hotplate. A 1.2-mm biopsy punch was then used to create the remaining ports in the bonded assembly of the upper layer and membrane. The membrane side of the assembly was then bonded to the lower PDMS layer by means of oxygen plasma treatment and baked at 90 °C for 15 min on a hotplate. The higher temperature ensured that sufficient heat reached the bonding surfaces through the lower PDMS layer.

### Device setup and testing

All devices were primed by submerging the device under distilled water and applying a vacuum of approximately 75 kPa below atmosphere for 10 min. Air was then slowly released into the vacuum chamber while the devices were submerged, priming the channels (including dead-ends) with distilled water. After priming, data collection was carried out on a benchtop in room air. Unless otherwise specified, all fluidic connections were made with 0.03-inch-inner-diameter fluorinated ethylene propylene (FEP) tubing (1520XL, IDEX-HS) and PEEK fittings purchased from IDEX Health & Sciences. The various tubular fluidic resistors were made using 0.01-inch-inner-diameter FEP tubing (1527L, IDEX-HS). The specific resistor lengths and other component details for each circuit are provided in Extended Data Table 1. Computer-controlled pressure sources (LineUp FlowEZ, Fluigent) were used to supply pressures for characterization of the microfluidic devices. Unless otherwise specified, all reservoirs for the pressure sources (P-CAP, Fluigent) were filled with 1× phosphate-buffered saline (PBS; Gibco PBS, Fisher Scientific). All pressure measurements were made using Honeywell pressure sensors (ABPDRRV015PDAA5) and logged on a computer using MATLAB. All flow measurements were made using Sensirion flow meters (SLI-1000).

### Single-transistor characterization

**Extended Data Fig. 7:**
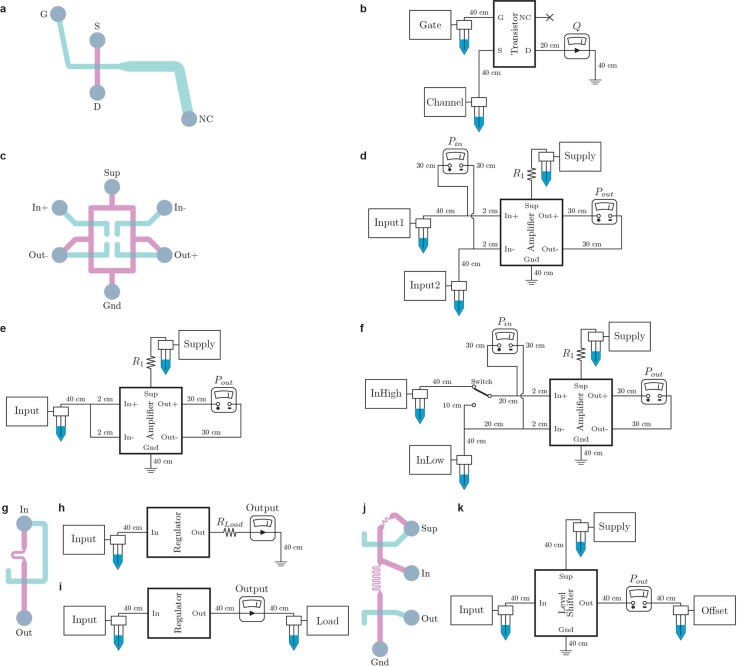
Setups for single transistor, amplifier, flow regulator, and level shifter measurements. Relevant component details such as geometry and resistance values are provided in Extended Data Table 1. **a**, Pinout diagram of single transistor chip (NC: no connection) with punched ports coloured gray, and channel layers coloured magenta and teal. **b**, Fluidic setup for single transistor characteristic curves and transconductance curve measurements. Cross-terminated link indicates no connection. **c**, Pinout diagram of amplifier chip with punched ports coloured gray, and channel layers coloured magenta and teal. **d**, Fluidic setup for amplifier demonstration and distortion measurements. **e**, Fluidic setup for amplifier common-mode rejection measurements. **f**, Fluidic setup for amplifier Frequency response (Bode plot) measurements. **g**, Pinout diagram of flow regulator chip with punched ports coloured gray, and channel layers coloured magenta and teal. **h**, Fluidic setup for regulator demonstration and line regulation measurements. **i**, Fluidic setup for load regulation measurements. **j**, Pinout diagram of level shifter chip with punched ports coloured gray, and channel layers coloured magenta and teal. **k**, Fluidic setup for level shifter demonstration, shift amount, and gain measurements.

The pinout for the single transistor chip is given in Extended Data Fig. 7a. Extended Data Fig. 7b provides the setup used to measure the transistor characteristic curves (Fig. 1c and Extended Data Fig. 1a). The ‘Gate’ pressure source and the ‘Channel’ pressure source used a Fluigent LU-FEZ-2000 module and a Fluigent LU-FEZ-1000 module respectively to control the pressure. To apply a given *P*_SD_ and *P*_GS_ to the device, the pressure at ‘Channel’ was set to *P*_SD_ and the pressure at ‘Gate’ was set to *P*_GS_ *+* *P*_SD_. To generate the characteristic curves, *P*_GS_ was set to 0 kPa, *P*_SD_ was swept from 0 kPa to 80 kPa over the course of 600 s, and the flow *Q* was recorded to generate each curve. Then, *P*_GS_ was incremented by 5 kPa, and the process was repeated until *P*_GS_ reached 80 kPa.

To obtain the intrinsic gain contour plot (Fig. 1d), the two-dimensional surface of points collected from the previous characteristic curve measurements was smoothed using a two-variable rational polynomial function of degree one in the numerator and degree two in the denominator. The smoothed polynomial was confirmed to fit the raw data well (*R*^2^ > 0.99) and was used to avoid noise when computing the numerical derivatives. The intrinsic gain was then calculated in MATLAB from the smoothed data (equation (3)). The smoothed data were also used to calculate the output impedance (Extended Data Fig. 1c) using equation (2) and the transconductance (Extended Data Fig. 1d) using equation (1).

The same setup (Extended Data Fig. 7b) was used to measure the transistor transfer characteristics (Extended Data Fig. 1b). To generate the transfer characteristic curves, *P*_SD_ was set to 20 kPa, *P*_GS_ was swept from 0 kPa to 80 kPa over the course of 300 s, and the flow *Q* was recorded to generate each curve. Then, *P*_SD_ was incremented by 20 kPa, and the process was repeated until *P*_SD_ reached 80 kPa.

### Amplifier characterization

The pinout for the amplifier is given in Extended Data Fig. 7c. Extended Data Fig. 7d provides the setup used to demonstrate the amplifier (Fig. 2a). The ‘Supply’ pressure source used a Fluigent LU-FEZ-7000 module to control the pressure. The ‘Input1’ and ‘Input2’ pressure sources used two Fluigent LU-FEZ-2000 modules. The tubing dimensions used for the resistances are provided in Extended Data Table 1. The ‘Supply’ pressure source was set to 250 kPa. The ‘Input1’ and ‘Input2’ pressure sources applied a common-mode bias of 175 kPa and a differential sinusoidal signal of amplitude 1 kPa and a period of 10 s. The differential input and output signals were measured by pressure sensors.

The same setup (Extended Data Fig. 7d) was used to measure the amplifier distortion (Extended Data Fig. 2a). The ‘Supply’ pressure source was set to 250 kPa. Over the course of 150 s, the ‘Input1’ pressure source was swept from 180 kPa to 170 kPa and the ‘Input2’ pressure source was swept from 170 kPa to 180 kPa. The differential input and output signals were measured by pressure sensors.

Extended Data Fig. 7e provides the setup used to measure the amplifier common-mode rejection (Extended Data Fig. 2b). The ‘Supply’ and ‘Input’ pressure sources used a Fluigent LU-FEZ-7000 and a Fluigent LU-FEZ-2000 module respectively to control the pressure. The tail resistance (*R*_1_) was fabricated using 30 cm of 0.01-inch-diameter FEP tubing (1527L, IDEX-HS). The ‘Supply’ pressure source was set to 250 kPa and the ‘Input’ pressure source was swept from 160 kPa to 200 kPa over the course of 150 s. The differential output signal was measured by a pressure sensor.

Extended Data Fig. 7f provides the setup used to determine the amplifier frequency response (Extended Data Fig. 2c). The ‘Supply’ pressure source used a Fluigent LU-FEZ-7000 module to control the pressure. The ‘InHigh’ and ‘InLow’ pressure sources used two Fluigent LU-FEZ-2000 modules. The ‘Switch’ was a Fluigent 2-switch (2SW002). The tail resistance (*R*_1_) was made using 30 cm of 0.01-inch-diameter FEP tubing (1527L, IDEX-HS). The ‘Supply’ pressure source was set to 250 kPa, the ‘InLow’ pressure source was set to 175 kPa, and the ‘InHigh’ pressure source was set to 177 kPa. The ‘Switch’ was set to toggle every 15 s. The differential input and output signals were measured by pressure sensors and data were collected over 500 s.

To generate the frequency response plot of the amplifier (Extended Data Fig. 2c), the differential input and output signals were resampled to a constant sampling frequency, and then converted to the frequency domain. As a square-wave excitation signal in the time domain produces only odd harmonics in the frequency domain, the first 40 odd harmonics of the input and output frequency-domain signals were used to generate the frequency response plot points.

### Flow regulator characterization

The pinout for the regulator chip is given in Extended Data Fig. 7g. Extended Data Fig. 7h provides the setup used to demonstrate the flow regulator (Fig. 2b). The ‘Input’ pressure source used a Fluigent LU-FEZ-2000 module to control the pressure. The *R*_load_ resistance was made using 20 cm of 0.01-inch-diameter FEP tubing (1527L, IDEX-HS). To simulate a poorly regulated pressure source, the ‘Input’ pressure source applied an arbitrary randomly generated pressure waveform ranging from approximately 75 kPa to 150 kPa over the course of 50 s while the flow through the load was recorded.

The same setup (Extended Data Fig. 7h) was used to measure the line regulation of the flow regulator (Extended Data Fig. 3a). The *R*_load_ resistance was made using 20 cm of 0.01-inch-diameter FEP tubing (1527L, IDEX-HS). The ‘Input’ pressure source was swept from 0 kPa to 150 kPa over the course of 300 s and the flow was recorded.

Extended Data Fig. 7i provides the setup used to measure the load regulation of the flow regulator (Extended Data Fig. 3b). The ‘Line’ and ‘Load’ pressure sources used Fluigent LU-FEZ-2000 modules to control the pressures. The ‘Line’ pressure source was set to 100 kPa. The ‘Load’ pressure source was swept from 0 kPa to 50 kPa over the course of 300 s and the flow was recorded.

### Level shifter characterization

The pinout for the level shifter chip is given in Extended Data Fig. 7j. Extended Data Fig. 7k provides the setup used to demonstrate the level shifter (Fig. 2c). The ‘Supply’ and ‘Input’ pressure sources used a Fluigent LU-FEZ-7000 and a Fluigent LU-FEZ-2000 module respectively to control the pressure. The ‘Offset’ pressure source was used to offset the pressure measurement and ensure an appropriate measurement range for the pressure sensor. The ‘Supply’ pressure source was set to 250 kPa, and the ‘Offset’ pressure source was set to 150 kPa. The ‘Input’ pressure source generated a sinusoidal waveform with an amplitude of 20 kPa, a baseline bias pressure of 80 kPa and a period of 30 s. The output pressure waveform was recorded using a pressure sensor and plotted over 150 s (five periods).

The same setup (Extended Data Fig. 7k) was used to measure the level shifter shift amount and gain (Extended Data Fig. 3c,d). The ‘Supply’ pressure source was set to 250 kPa, and the ‘Offset’ pressure source was set to 150 kPa. The ‘Input’ pressure source was swept from 10 kPa to 90 kPa over the course of 240 s and the output pressure was recorded. The shift amount was determined by subtracting the output pressure from the pressure applied at the ‘Input’ pressure source. The output pressure data were smoothed using a polynomial function of degree three to remove measurement noise, and then the gain was calculated from the derivative. Note that this circuit operates in a common-drain configuration, and so the pressure gain is expected to be less than unity.

### NAND gate characterization

**Extended Data Fig. 8:**
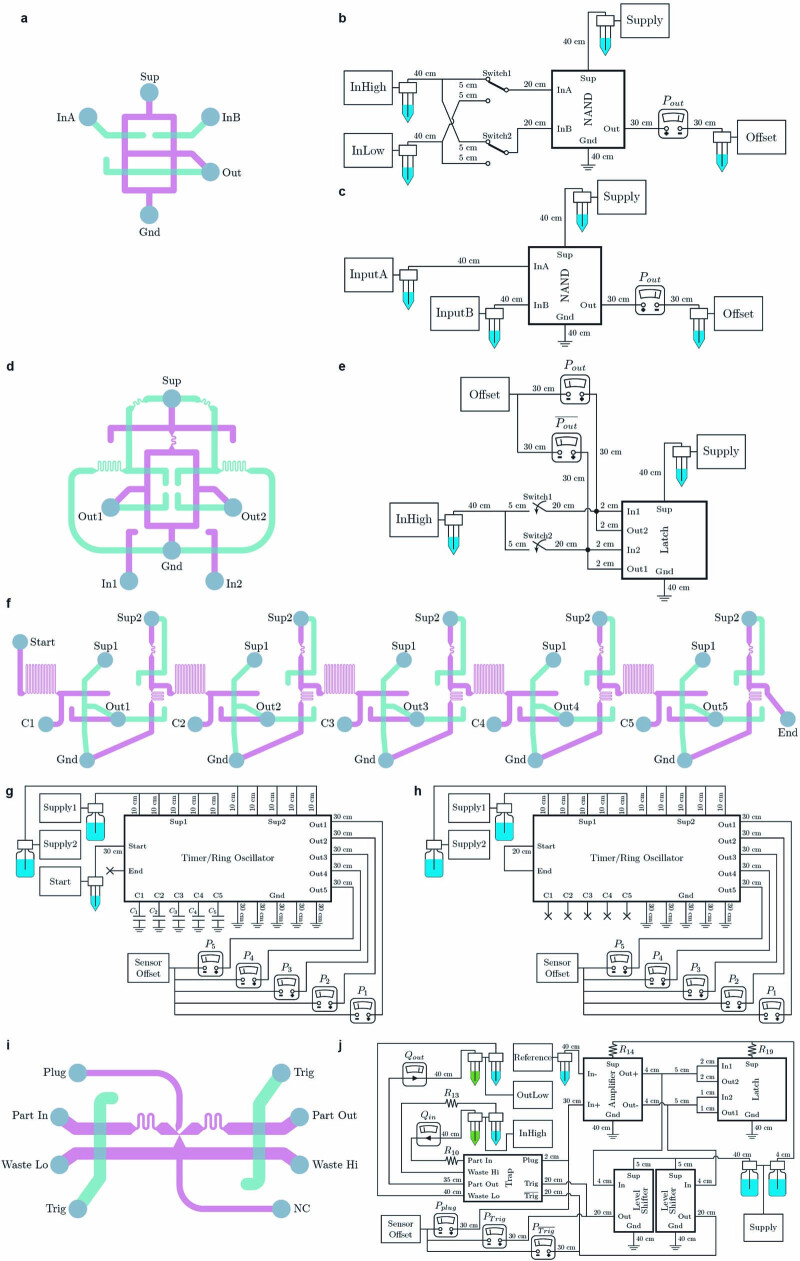
Setups for NAND gate, SR-latch, timer, ring oscillator, and smart particle dispenser measurements. Relevant component details such as geometry and resistance values are provided in Extended Data Table 1. **a**, Pinout diagram of NAND gate chip with punched ports coloured gray, and channel layers coloured magenta and teal. **b**, Fluidic setup for NAND gate demonstration and output dynamics measurements. **c**, Fluidic setup for NAND gate transfer characteristics measurements. **d**, Pinout diagram of SR-latch chip with punched ports coloured gray, and channel layers coloured magenta and teal. **e**, Fluidic setup for SR-latch demonstration and response dynamics measurements. **f**, Pinout diagram of timer/oscillator chip with punched ports coloured gray, and channel layers coloured magenta and teal. **g**, Fluidic setup for timer demonstration. Cross-terminated link indicates no connection. **h**, Fluidic setup for ring oscillator demonstration and jitter measurements. Cross-terminated link indicates no connection. **i**, Pinout diagram of particle trap chip (NC: no connection) with punched ports coloured gray, and channel layers coloured magenta and teal. **j**, Fluidic setup for particle dispenser ordering and concentration. Several blocks are used for signal processing. The Supply sources used 500 ml bottles as reservoirs. Green reservoirs hold bead suspensions. In this configuration the Trig and Sense signals are directly connected to each other to perform particle ordering and concentration.

The pinout for the NAND gate is given in Extended Data Fig. 8a. Extended Data Fig. 8b provides the setup used to demonstrate the NAND gate (Fig. 2d). The ‘Supply’ pressure source used a Fluigent LU-FEZ-7000 module to control the pressure. The ‘InHigh’ and ‘InLow’ pressure sources used two Fluigent LU-FEZ-2000 modules. The ‘Offset’ pressure source used a Fluigent LU-FEZ-1000. ‘Switch1’ and ‘Switch2’ were Fluigent 2-switches (2SW002). The ‘Supply’ pressure source was set to 150 kPa, the ‘Offset’ pressure source was set to 100 kPa, the ‘InLow’ pressure source was set to 125 kPa, and the ‘InHigh’ pressure source was set to 175 kPa. Both ‘Switch1’ and ‘Switch2’ were set to toggle every 2.5 s, resulting in two square-wave pressure signals with a period of 5 s. The switches were timed such that the two pressure waveforms had a 1.25-s phase delay between them. The output pressure signal was recorded over the course of 300 s.

The same setup (Extended Data Fig. 8b) was used to measure the NAND gate output dynamics (Extended Data Fig. 4a,b), revealing the maximum rate of change in the circuit output. The ‘Supply’ pressure source was set to 150 kPa, the ‘InLow’ pressure source was set to 125 kPa, and the ‘InHigh’ pressure source was set to 175 kPa. ‘Switch1’ was set to toggle every 2.5 s, while ‘Switch2’ was maintained in the top position, connecting the ‘InB’ port to the ‘InHigh’ pressure source. The output pressure signal was recorded over the course of 300 s. Fifty-five individual rising and falling edges were overlaid and plotted.

Extended Data Fig. 8c provides the setup used to measure the NAND gate transfer characteristics (Extended Data Fig. 4c,d). The ‘Supply’ pressure source used a Fluigent LU-FEZ-7000 module to control the pressure. The ‘InputA’ and ‘InputB’ pressure sources used two Fluigent LU-FEZ-2000 modules. The ‘Offset’ pressure source used a Fluigent LU-FEZ-1000. The ‘Supply’ pressure source was set to 150 kPa, and the ‘Offset’ pressure source was set to 100 kPa. To measure the Input A transfer characteristics (Extended Data Fig. 4c), the ‘Input A’ pressure source was swept from 125 kPa to 175 kPa over the course of 15 s while ‘Input B’ was held high at 175 kPa. Subsequently, to measure the Input B transfer characteristics (Extended Data Fig. 4d), the ‘Input B’ pressure source was swept from 175 kPa to 125 kPa over the course of 15 s while ‘Input A’ was held high at 175 kPa. The output pressure signal was recorded as these sweeps were repeated ten times each. These transfer characteristics were overlaid and plotted.

### SR latch characterization

The pinout for the SR latch is given in Extended Data Fig. 8d. Extended Data Fig. 8e provides the setup used to demonstrate the SR latch (Fig. 2e). The ‘Supply’ pressure source used a Fluigent LU-FEZ-7000, the ‘InHigh’ pressure source used a Fluigent LU-FEZ-2000, and the ‘Offset’ pressure source used a Fluigent LU-FEZ-1000. ‘Switch1’ and ‘Switch2’ were Fluigent 2-switches (2SW002) normally in the open state. The ‘Supply’ pressure source was set to 250 kPa, the ‘InHigh’ pressure source was set to 165 kPa, and the ‘Offset’ pressure source was set to 100 kPa. The latch was set by briefly closing and reopening ‘Switch1’ for the shortest period the Fluigent SDK would allow (0.5 s). The latch was then reset by briefly closing and reopening ‘Switch2’ for the shortest period the Fluigent SDK would allow. To demonstrate the memory of the latch (Fig. 2e), the output pressures were recorded as it was set and reset with arbitrarily varying time intervals between the set and reset operations.

The same setup (Extended Data Fig. 8e) was used to measure the SR latch set and reset response (Extended Data Fig. 4e,f), revealing the response dynamics and speed of the circuit. The ‘Supply’ pressure source was set to 250 kPa, the ‘InHigh’ pressure source was set to 165 kPa, and the ‘Offset’ pressure source was set to 100 kPa. The set and reset operations were carried out by briefly closing the switches as described above. In this fashion, the latch was alternatively set and reset every 2.5 s while the output pressures were measured over the course of 300 s. The resulting pressure signal consisted of sixty reset output edges (Extended Data Fig. 4e) and sixty set complementary edges (Extended Data Fig. 4f).

### Timer characterization

The pinout for the timer is given in Extended Data Fig. 8f. Extended Data Fig. 8g provides the setup used to demonstrate the timer (Fig. 3b). The timer uses two different power supplies for the amplifiers and the level shifters of the inverters. Each set of power supply lines from the chip leads to a power supply bus line made of luer-lock T-junctions. The large diameter of the power supply bus lines reduces fluidic resistance, providing a constant-pressure source to all of the components on the microfluidic chip. In total, running the whole five-stage chip consumes approximately 50 μl s^−1^ of liquid for power. The ‘Supply2’ pressure source used a Fluigent LU-FEZ-7000 module to control the pressure. The ‘Supply1’ and ‘Start’ pressure sources used two Fluigent LU-FEZ-2000 modules. The ‘Offset’ pressure source used a Fluigent LU-FEZ-1000.

The timer circuit uses off-chip fluidic capacitors to easily change the intervals timed out by the chip, although any construction of fluidic capacitors should work equivalently. The fluidic capacitors used here were 1-ml syringes filled with different fixed volumes of air, whose effective fluidic capacitance is calculated using Boyle’s law and the initial volume of air (values provided in Extended Data Table 1). The air-syringe capacitors were created by simply withdrawing the plunger in air to a certain volume, then gluing the plunger in place. The different air volumes used in the five syringes exhibit different fluidic capacitances and therefore time out different intervals.

To demonstrate the timer (Fig. 3b), the ‘Supply1’ pressure source was set to 160 kPa, the ‘Supply2’ pressure source was set to 200 kPa, and the ‘Offset’ pressure source was set to 100 kPa. The ‘Start’ pressure source was initially set to 140 kPa, and then was set to 180 kPa after 300 s, triggering the start of the timer. The signal then propagated through the circuit, triggering step responses in the measured output pressure signals *P*_1_ to *P*_5_ at fixed intervals in time. The output signals were recorded over 120 s. The results of three separate runs of the timer chip were overlaid and plotted in Fig. 3b, showing good repeatability.

### Ring oscillator characterization

The pinout for the ring oscillator is also given in Extended Data Fig. 8f. Extended Data Fig. 8h provides the setup used to demonstrate the ring oscillator (Fig. 3d). The setup for the oscillator is similar to that of the timer circuit, using the same power supply bus lines and pressure sensors. However, the capacitors were removed and replaced by fluidic plugs (no connection), and the ‘Finish’ pin was fed back and connected to the ‘Start’ pin, forming a loop. Like with the timer, the ‘Supply2’ pressure source used a Fluigent LU-FEZ-7000 module to control the pressure. The ‘Supply1’ pressure source used a Fluigent LU-FEZ-2000 module. The ‘Offset’ pressure source used a Fluigent LU-FEZ-1000. To demonstrate the oscillator (Fig. 3d), the ‘Supply1’ pressure source was set to 160 kPa, the ‘Supply2’ pressure source was set to 200 kPa, and the ‘Offset’ pressure source was set to 100 kPa. Following power-up, the circuit spontaneously began oscillating. The period square-wave output signals from the inverters were recorded for 300 s. The data from the first 30 s as the circuit was powering up were discarded, and the remaining signals were split into individual periods referenced by the rising edge of *P*_1_ crossing a threshold of 80 kPa (halfway between the high and low logic levels). These periods (63 from each of the 5 signals) were overlaid and plotted in Fig. 3d to create an eye diagram of the inverters in the oscillator ring. The jitter plot (Extended Data Fig. 5a) for the oscillator depicts a histogram of the time delay between the threshold crossing time of *P*_1_ and that of each of the subsequent inverter signals, each separated by one-fifth the period.

### Smart particle dispenser characterization

The function of each of the circuit blocks in the smart particle trap is described below. When a particle is trapped, the pressure upstream of the trap (*P*_plug_) rises slightly. An amplifier circuit block is used to amplify this small change and compare it with a reference threshold pressure, producing a pair of complementary signals indicating the presence of a particle. The latch circuit block ensures complementarity of the signals and also acts to suppress any spurious noise events that were amplified. Finally, these signals are shifted up using level shifter circuit blocks to produce the output Sense and complementary (signified by an overbar) $\overline{{\rm{Sense}}}$ signals. The complementary Trig and $\overline{{\rm{Trig}}}$ signals are used to control the direction of flow in the trap.

The concentration and ordering capabilities of the smart particle dispenser circuit were tested using a suspension of polystyrene microspheres in PBS. The suspension was prepared by adding 40-μm-diameter polystyrene beads (Fluoro-Max Green 35-7B, Thermo-Fisher) to 50 ml of 1× PBS (Gibco PBS, Fisher Scientific) to achieve a final concentration of approximately 30 beads per millilitre.

The pinout for the particle trap is given in Extended Data Fig. 8i. Extended Data Fig. 8j provides the setup used to test the smart dispenser configured for particle concentration and ordering. The reservoir (green) connected to the ‘Part In’ line of the trap was filled with the dilute polystyrene bead suspension and all other reservoirs were filled with PBS. The reservoirs connected to the ‘Supply’ pressure source were 500-ml bottles, whereas all other reservoirs were P-CAP reservoirs from Fluigent. The ‘Supply’ pressure source used a Fluigent LU-FEZ-7000 module to control the pressure. The ‘InHigh’, ‘OutLow’ and ‘Reference’ pressure sources used Fluigent LU-FEZ-2000 modules to control the pressure. The ‘Sensor Offset’ pressure source used a Fluigent LU-FEZ-1000 module to offset the pressure sensors, ensuring an appropriate measurement range. The tubing dimensions used for the resistances are provided in Extended Data Table 1. The ‘Supply’ pressure source was set to 250 kPa, the ‘InHigh’ pressure source was set to 160 kPa, the ‘OutLow’ pressure source was set to 140 kPa, the ‘Reference’ pressure source was set to 150 kPa, and the ‘Sensor Offset’ pressure source was set to 100 kPa.

All pressure sources remained constant during the entirety of the experiment, as all of the dynamic signal processing was performed by the microfluidic chip itself. Trapping events were consistently detected by a sharp rising edge in the *P*_plug_ pressure signal, and additionally verified visually under a microscope. Between trapping events, the flow through the ‘Part In’ line (*Q*_in_) was integrated to compute the input particle spacing volume, and the flow through the ‘Part Out’ line (*Q*_out_) was integrated to compute the output particle spacing volume. The experiment was run for 230 trapping events before the ‘Supply’ reservoirs of liquid to power the system were depleted.

## Online content

Any methods, additional references, Nature Portfolio reporting summaries, source data, extended data, supplementary information, acknowledgements, peer review information; details of author contributions and competing interests; and statements of data and code availability are available at 10.1038/s41586-023-06517-3.

### Supplementary information

Supplementary Data 1 Vector file containing the top and bottom layer masks for all circuits. Note that when preparing the SU-8 wafer, one mask must be inverted, as each final device consists of one feature-up and one feature-down PDMS layer. Scale: 1 unit = 1 μm.

## Figures and Tables

**Extended Data Table 1:**
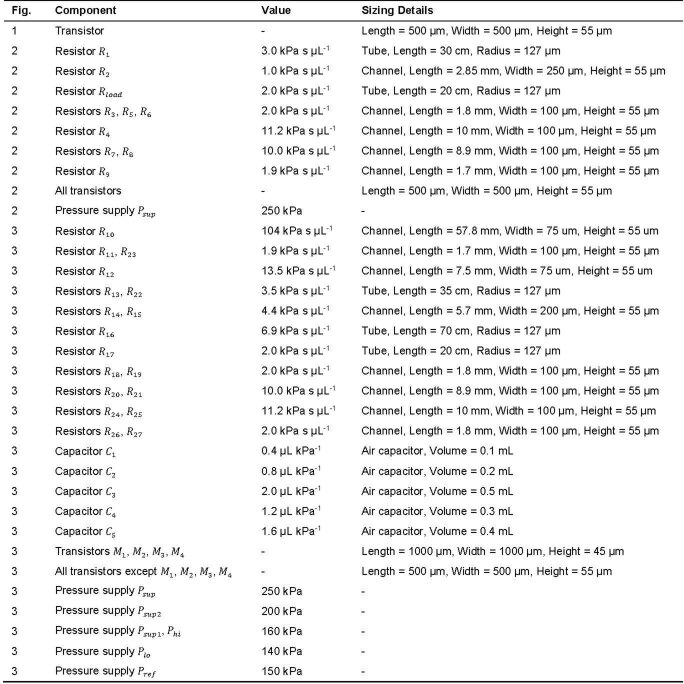
Circuit schematic component details

Specific values and sizes for the resistors, capacitors, transistors, and pressure sources used in the circuit schematics are provided. Resistors may be integrated on-chip using a serpentine channel (denoted “Channel”) with a rectangular cross-section, or incorporated into the tubing that leads to the chip ports (denoted “Tube”) with a circular cross-section. Resistances were calculated under laminar flow conditions using the Poiseuille equation^47^. 1 ml syringes filled with a fixed volume of air were used as capacitors to atmosphere. Fluidic capacitances were calculated using Boyle’s law. For more information, please see the Methods section.

**Extended Data Table 2:**
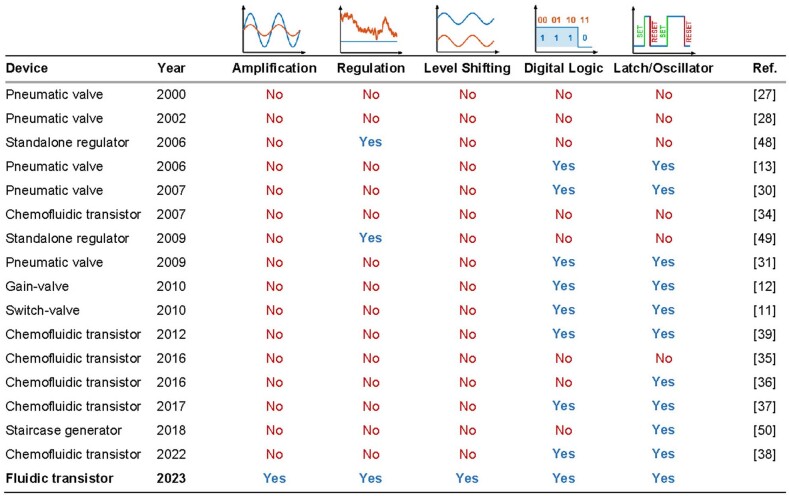
Summary of signal processing capabilities of microfluidic platforms for automatic control

The capabilities of several microfluidic technologies that operate on fluidic (pressure/flow) signals are summarized. Chemofluidic elements use a combination of chemical and fluidic signal processing, but may be implemented to make fluidic logic, latches, and oscillators. The first three operations shown (amplification, regulation, and level shifting) belong to analog signal processing, whereas the remaining two (digital logic and memory) belong to digital signal processing. Microfluidic platforms that demonstrate the full suite of building-block capabilities can be used to translate a wide range of more complex electronic circuits into the microfluidic domain. References ^48–50^.

## Data Availability

All relevant data are included in the article and/or Supplementary Data 1. A preprint of this paper is available at *bioRxiv*^46^.

## Code availability

No specialized code or algorithms were used to analyse the data in the current study.
